# Bacterial Contaminants in Ambulances from a Tertiary Care Hospital as Potential Threats to Patients and Medical Staff in Al-Qassim Region, Saudi Arabia—Effect of Decontamination

**DOI:** 10.3390/pathogens14121301

**Published:** 2025-12-18

**Authors:** Ahmed E. Taha, Ahmad R. Alharbi, Omar N. Alharbi, Alaaeldin M. Komila, Abdullah Almushawwah, Solaiman Aldeghaim, Ahmed N. Algefary, Majed Allahim, Khalid Alzaben, Faisal M. Alharbi

**Affiliations:** 1 Microbiology and Immunology Unit, Department of Pathology, College of Medicine, Jouf University, Sakaka City 72388, Aljouf, Saudi Arabia; 2 Department of Medical Microbiology and Immunology, Faculty of Medicine, Mansoura University, Mansoura City 35516, Aldakahlia Governorate, Egypt; 3 Radiology Department, Alqwarah General Hospital, Ministry of Health (MOH), Buraydah City 51452, Alqassim, Saudi Arabia; ahraalharbi@moh.gov.sa; 4 Infection Prevention and Control, College of Medicine, Jouf University, Sakaka City 72388, Aljouf, Saudi Arabia; 5 Emergency Medical Services Department, Alqwarah General Hospital, Ministry of Health (MOH), Buraydah City 51452, Alqassim, Saudi Arabia; omarna@moh.gov.sa; 6 Department of Medical Laboratories, College of Applied Medical Sciences, Qassim University, Buraydah City 51452, Alqassim, Saudi Arabia; a.komila@qu.edu.sa (A.M.K.); abd.almushawwah@qu.edu.sa (A.A.); so.aldeghaim@qu.edu.sa (S.A.); ah.algefary@qu.edu.sa (A.N.A.); m.allahim@qu.edu.sa (M.A.); 7 Administration of Forensic Toxicology Services, Forensic Medical Services Center, Ministry of Health (MOH), Buraydah City 51452, Alqassim, Saudi Arabia; kalzben@moh.gov.sa; 8 Infection Control Directorate at the Public Health Authority Office, Ministry of Health (MOH), Buraydah City 51452, Alqassim, Saudi Arabia

**Keywords:** ambulance, antibiotic, disinfection, healthcare-associated infections, multidrug-resistant organism, nosocomial, prevention strategies, resistance

## Abstract

Bacterial contaminants in ambulances could have a major impact on morbidities, mortalities, and healthcare resources, especially if these bacteria are antimicrobial-resistant. As far as we know, this is the first study in Al-Qassim region to evaluate the prevalence of bacterial contaminants in swab samples obtained from ambulances from Alqwarah General Hospital, Al-Qassim region, Saudi Arabia as an indicator for evaluation of the implemented infection control measures, and screen the antibiotics profiles of the isolates against the most regularly used antimicrobials. In total, 204 samples were collected from the ambulances following patient transport. To evaluate the effect of vehicle decontamination, 204 swabs were collected from the same sites of the ambulances immediately after cleaning and disinfection. The isolates were identified using standard bacteriological and biochemical methods, as recommended by the Clinical Laboratory Standard Institute (CLSI). The antibiotic susceptibility patterns were assessed using the Kirby–Bauer disc diffusion method. The prevalence of bacterial contamination in the samples collected following patient transport was 46.08%. In total, 83.33%, 75.00%, and 66.66% of the samples collected from DC shock apparatuses, ceilings, and emergency personnel seats, respectively, were contaminated. Furthermore, ceilings, DC shock apparatuses, emergency personnel seats, cervical collars, and monitors were found to harbor 10.8%, 9.8%, 7.8%, 6.8%, and 6.8% of the 102 bacterial isolates, respectively. Gram-positive organisms represented 96.1% of all bacterial isolates. *Bacillus* spp. was the most common isolate, accounting for 60.8% of all bacterial isolates. Although *Pseudomonas aeruginosa* and *Proteus* spp. isolates were sensitive to all the tested antimicrobials, many Gram-positive bacterial isolates were resistant to some antibiotics in variable frequencies. After 48 h of aerobic incubation (with or without 5–10% CO_2_) on nutrient, blood, chocolate, and MacConkey agar plates at 37 °C, no bacterial growth was detected in the samples collected immediately following cleaning and disinfection. This is the second Saudi study to evaluate the prevalence of bacterial contaminants in Saudi Arabian ambulances, and it could help health policy makers in improving the implemented infection prevention and control measures in Saudi Arabian ambulances. The samples taken after patient transport revealed bacterial contaminants with varying rates of antimicrobial resistance. Policies ensuring the optimal cleaning and disinfection of ambulances can minimize the potential of bacterial infection for high-risk patients, their relatives, and healthcare providers.

## 1. Introduction

The ambulances utilized by emergency medical care teams are workplaces for qualified medical and paramedical staff who oversee daily transportation for patients in life-threatening conditions from all walks of life with a variety of infections, diseases, and injuries. Millions of seriously ill and injured patients are transported to and from hospitals and other healthcare (HC) facilities by emergency medical services (EMS) personnel, who are frequently referred to as the “front lines” of the HC system. EMS providers frequently encounter a wide range of hazardous and erratic circumstances while carrying out their jobs, endangering both their own and their patients’ safety. EMS personnel are up to seven times more likely to suffer harm at work [1].

Numerous surfaces, including the patient-care compartments of ambulances, around the world have been found to harbor the organisms most frequently linked to healthcare-associated infections (HAIs). The complexity of addressing the accompanying infections makes organisms detected in ambulance environments problematic, especially if they are of a multidrug resistant (MDR) nature. Emergency care frequently involves people in life-threatening situations. When using ambulance services, vulnerable groups such as the old, young, immunocompromised, and seriously injured are frequently treated concurrently. Therefore, transport vehicles may be potential sources of transmission of microbes to high-risk patients, their relatives, and HC providers [2]. By encountering infected patients and their body fluids, as well as by visiting a variety of locations throughout their shifts (such as homes, workplaces, and hospitals), EMS providers are probably regularly exposed to biohazard threats from microorganisms [3].

According to the World Health Organization (WHO), millions of HAIs occur annually, affecting around 7% and up to 19% of patients in developed and developing countries, respectively [4]. According to the American Centers for Disease Control and Prevention (CDC), many bacteria, viruses, and/or fungi can cause HAIs, but special attention has been paid to the organisms that are highly contagious, exhibit high virulence, or are resistant to common antimicrobials. MDR bacteria are of particular concern because they can cause serious and challenging-to-treat HAIs [5]. Bacterial contaminants in ambulances could have a major impact on morbidities, mortalities, and HC resources, especially if these bacteria are MDR. Thus, ambulances may pose a risk of serious infection to patients, their relatives, HC providers, and paramedics [2,3].

Bacterial contamination of ambulances has been the subject of numerous studies worldwide [6,7,8,9,10,11,12,13,14,15,16,17,18,19,20,21,22,23,24,25]. *Bacillus* species (spp.) have been reported in many countries, including the UK [6], the USA [7], Saudi Arabia [14], and Spain [19]. In the UK, micrococci have been detected among ambulances’ bacterial contaminants [6]. In the UK [6], the USA [7], South Korea [10,12], Iraq [13], Spain [19], Denmark [20], Egypt [21], Iran [23], and India [24], Gram-negative bacterial contaminants have been isolated. Staphylococci were detected in UK [6], USA [7,8,11,22], Germany [9,16], South Korea [10,12], Saudi Arabia [14], Thailand [15], Australia [17], Denmark [18,20], Spain [19], Egypt [21], Iran [23], India [24], and Poland [25].

The family Firmicutes contains the genus *Bacillus*, which is composed of aerobic, facultative anaerobic, Gram-positive spore-forming bacilli with low GC content. The amount of vegetation, mean annual temperature, soil moisture content, and pH all have an impact on the abundance of *Bacillus* spores in the soil. Numerous pathogenic and non-pathogenic *Bacillus* spp. are frequently found in soil [26]. Micrococci are spherical, Gram-positive, coagulase-negative, catalase-positive cocci that are frequently seen in tetrads and are members of the Micrococcaceae family. *Micrococcus* species are frequently found in the environment and can cause opportunistic infections in people with impaired immune systems [27]. *Moraxella* is an aerobic, oxidase-positive, Gram-negative diplococcus that is often considered a potentially pathogenic genus, particularly in immunocompromised people [28]. The prevalence of bacteria, including staphylococci, in ambulances highlights the importance of maintaining high hygiene and decontamination standards during regular prehospital work with patients [6].

Infection or colonization with MDR organisms found in ambulances increases the probability of complications. Given the frequency of organisms often linked to HAIs in the patient-care compartments of ambulances, it is obvious that the best decontamination compliance procedures are not always followed [2]. The contribution of EMS vehicles and equipment to the epidemiological transmission of HAIs both inside and outside the HC industry requires further research. As far as we are aware, there is just one study looking at the bacterial contamination of Saudi Arabian ambulances [14]. In this prospective, our study aims were to determine the prevalence of bacterial contaminants in the ambulances used at Alqwarah General Hospital in the Al-Qassim region of Saudi Arabia and to assess the isolates’ antibiotic profiles against the most-used antimicrobials as indicators for evaluating the effectiveness of the infection control strategies that were put in place.

## 2. Materials and Methods

### 2.1. Design of the Study

Bioethical approval (no. 607-46-010690) was obtained from the Qassim Region Research Ethics Committee (QREC; No. H-04-Q-001), Qassim, Saudi Arabia. According to the inclusion criteria, we included ambulances that were regularly used for transfer of patients, ambulances whose drivers were unaware of when sampling visits would be conducted, and ambulances whose drivers agreed to cooperate. We excluded ambulances that were out of service, ambulances whose drivers previously knew when sampling visits would be conducted, and ambulances whose driver refused to cooperate.

Swab samples were collected from different sites of two ambulances at Alqwarah General Hospital, Al-Qassim region, Saudi Arabia, in a cross-sectional study during April and May 2025. The privacy and confidentiality of those responsible for the ambulances were protected. Processing of the samples was performed in the microbiology laboratory of the Department of Medical Laboratories, College of Applied Medical Sciences, Qassim University, Buraydah City 51452, Saudi Arabia, after obtaining the required permissions.

### 2.2. Collection and Transport of Samples

The required sample size for each of the two ambulances was estimated using the following formula: *n* = (*Z*^2^ × *p*(1 − *p*))/*d*^2^, where *n* = sample size, *Z* = *Z*-score corresponding to the desired confidence level (*Z* = 1.96 when confidence level = 95%), *p* = the estimated proportion of contamination (a conservative estimate was used: *p* = 0.5), and *d* = desired margin of error (we used a margin of error of 0.1) [29]. Thus, the estimated sample size for each ambulance was 96 swabs. In total, 204 samples were collected from different sites of two ambulances following patient transport (17 swabs were collected from each ambulance in six sampling visits).

We used sterile cotton-tipped swabs with amies transport media (GlobalRoll^®^, Hangzhou, China). The sampling sites were as follows: carrying handle of cart, sidebar of cart, DC shock apparatus, stethoscope, cervical collar, inside wall of vehicle, emergency personnel seats, portable ventilator, blood pressure cuff, monitor, door grip, oxygen humidifier glass, suction device, headboard of patient stretcher, and steering wheel. The sterile swabs, which had been moistened with the transport media, were rolled over all the selected surfaces.

To evaluate the effect of vehicle decontamination, 204 swabs were collected from the same sites of the ambulances immediately after cleaning and disinfection. Standard bleach (sodium hypochlorite solution, 10,000 parts per million; normal dilution, 1:10) was the main disinfectant that was used, especially for blood spills and external surfaces. Alcohol (70–95%) was used for external surfaces of certain pieces of equipment, including stethoscopes and pulse oximeters. For walls, floors, and furnishings, quaternary ammonium compounds or hydrogen peroxide was used.

Each sample was collected in a sterile bag and carried in an icebox to be processed according to the standard microbiological methods in the microbiology laboratory of the Department of Medical Laboratories, College of Applied Medical Sciences, Qassim University after the required permissions were obtained.

### 2.3. Isolation and Identification of Bacteria

To avoid contamination, the samples were handled aseptically upon arrival. All media used in this study were prepared according to the manufacturer’s guidelines. The swabs were placed in tubes containing five milliliters of double-strength brain–heart infusion broth (Oxoid, Hampshire, UK) and incubated (with or without 5–10% CO_2_) aerobically at 37 °C for 24 h. After enrichment, the swabs were cultured on nutrient, blood, chocolate, and MacConkey agar plates (Oxoid, Hampshire, UK) and incubated (with or without 5–10% CO_2_) aerobically at 37 °C for an additional 48 h. To isolate pure single colonies of each bacterial isolate, the colonies were further subcultured on these agar plates under the same conditions for another 24 h. Following this, the pure colonies were analyzed using standard microbiological techniques, which included examining colony morphology (size, color, and shape), performing Gram staining, and conducting biochemical tests [30].

### 2.4. Antibiotic Sensitivity Testing

The antibiotic susceptibilities patterns of the bacterial isolates were assessed via the Kirby–Bauer disc diffusion method according to the guidelines of the clinical and laboratory standards institute (CLSI) [31]. The Gram-positive bacterial isolates were tested against the following antimicrobials: AP—Ampicillin, 10 μg; AK—Amikacin, 30 μg; AUG—Augmentin, 30 μg; BA—Bacitracin, 10 U; C—Chloramphenicol, 30 μg; CAZ—Ceftazidime, 30 μg; CIP—Ciprofloxacin, 5 μg; CD—Clindamycin, 2 μg; CPM—Cefepime, 30 μg; E—Erythromycin, 15 μg; FOX—Cefoxitin, 30 μg; GM—Gentamicin, 10 μg; Imipenem, 10 μg; KF—Cephalothin, 30 μg; NE—Neomycin, 30 μg; OT—Oxytetracycline, 30 μg; OX—Oxacillin, 5 μg; PG—Penicillin G, 10 U; PRL—Piperacillin, 30 μg; T—Tetracycline, 30 μg; TS—Cotrimoxazole, 25 μg; and VA—Vancomycin, 30 μg. The Gram-negative bacterial isolates were tested against the following antimicrobials: AP—Ampicillin, 10 μg; AK—Amikacin, 30 μg; ATM—Aztreonam, 30 μg; AUG—Augmentin, 30 μg; C—Chloramphenicol, 30 μg; CAZ—Ceftazidime, 30 μg; CIP—Ciprofloxacin, 5 μg; CPM—Cefepime, 30 μg; FOX—Cefoxitin, 30 μg; GM—Gentamicin, 10 μg; Imipenem, 10 μg; KF—Cephalothin, 30 μg; NE—Neomycin, 30 μg; PRL—Piperacillin, 30 μg; PB—Polymyxin B, 300 U; and TS—Cotrimoxazole, 25 μg.

### 2.5. Quality Control (QC)

ATCC strains (*Staphylococcus aureus*; ATCC25923 as QC for Gram-positive isolates; and *Escherichia coli* ATCC10536 as QC for Gram-negative isolates) were used as positive controls. QC testing was carried out in triplicate. Each isolate was tested in triplicate. The study’s negative controls for bacterial culture were non-inoculated nutrient, blood, chocolate, and MacConkey agar plates incubated (with or without 5–10% CO_2_) aerobically at 37 °C for 48 h. All data were evaluated using CLSI’s guidelines [31].

### 2.6. Data Analysis

Categorical data was presented as numbers and percentages. The total bacterial contamination prevalence rate was determined from the number of growth-positive swabs at the time the study was conducted divided by the number of all collected swabs. Each sampling site’s bacterial contamination prevalence was tested statistically by using the Chi-square (*_X_*^2^) test to compare the likelihood of an event (bacterial contamination) occurring between two groups (growth-positive/growth-negative swabs). Alternatively, the Fisher Exact (^FE^) correction test was applied when more than 20% of the cells had an expected count of less than 5. Statistical significance was considered at *p* ≤ 0.05.

## 3. Results

During the study, different samples were collected from the ambulances used by Alqwarah General Hospital, Al-Qassim region, Saudi Arabia. A total of 204 samples were collected following patient transport. Under suitable incubation conditions, samples were processed and grown on suitable media. Bacterial contaminants were detected (94 bacterial-growth-positive swabs/204 samples collected from the ambulances after the patients had been transported—46.08%). Out of the 94 swabs with positive bacterial growth, 86 samples revealed a single pathogen, while 8 samples revealed two pathogens. As a result, the overall count of bacterial isolates was 102.

The frequency of positive bacterial growth from each site of each vehicle is presented in Table 1. It is clear that 83.33%, 75.00%, and 66.66% of the samples collected from DC shock apparatuses, ceilings, and emergency personnel seats, respectively, were contaminated. Statistically, it is clear that the DC shock apparatuses and ceilings have a greater probability of being contaminated (*p* ≤ 0.05). Furthermore, the frequency of bacterial isolates in each site of each ambulance is presented in Table 2. In total, 10.8%, 9.8%, 7.8%, 6.8%, and 6.8% of the bacterial isolates were recovered from the ceilings of the ambulances, DC shock apparatuses, emergency personnel seats, cervical collars, and monitors, respectively.

The distribution of bacterial isolates on each site in the ambulances is presented in Table 3. It is clear that Gram-positive organisms represent 96.1% of all the bacterial isolates (98 Gram-positive/102 total isolates). *Bacillus* spp. was the most common isolate, accounting for 60.8% of all the bacterial isolates (62 *Bacillus* spp./102 total isolates), followed by *Micrococcus* spp., which represented 30.4% of the total bacterial isolates. The Gram-negative isolates only represented 3.9% of the total bacterial isolates.

Regarding the antimicrobial resistance patterns of the Gram-positive bacterial isolates, variable frequencies of resistance to some antibiotics were detected, as presented in Table 4. The control strain *S. aureus* ATCC25923 was susceptible to the tested antimicrobial. The Gram-negative bacterial isolates were tested against many antimicrobials. Fifty percent of the *Moraxella* isolates were resistant to cotrimoxazole and cephalothin antimicrobials. The isolates of *Moraxella* were susceptible to the remaining antibiotics, while the *Pseudomonas aeruginosa* and *Proteus* spp. isolates were sensitive to all the tested antibiotics. The control strain, *E. coli* ATCC10536, was susceptible to the tested antimicrobials.

Concerning the samples collected from the ambulances immediately following cleaning and disinfection, no bacterial growth was detected after 48 h of aerobic incubation (with or without 5–10% CO_2_) on nutrient, blood, chocolate, or MacConkey agar plates blood agar plates at 37 °C.

## 4. Discussion

Bacterial contaminants found in ambulances must be further isolated and characterized to be properly prevented and controlled. In this prospective study, we aimed to determine the prevalence of bacterial contaminants in swab samples obtained from ambulances used by Alqwarah General Hospital, Al-Qassim region, Saudi Arabia as an indicator for evaluating the implemented infection prevention and control measures, and screen the isolates’ antibiotics profiles for the most regularly used antimicrobials. The bacterial contamination prevalence in the samples collected following patient transport was 46.08%. The ambulances’ ceilings, DC shock apparatuses, and emergency personnel seats were the most common sites for bacterial contaminant detection.

Variable bacterial contamination rates have been reported in many countries, including the United Kingdom, the USA, Germany, South Korea, Iraq, Saudi Arabia, Thailand, Australia, Denmark, Spain, Egypt, Iran, India, and Poland [6,7,8,9,10,11,12,13,14,15,16,17,18,19,20,21,22,23,24,25], with great variability in isolate types, and the highly contaminated sites are presented in Appendix A Table A1. To our knowledge, only one prior Saudi study has examined bacterial contamination in ambulances in Saudi Arabia. This research was carried out at the EMS Department of Prince Sultan Bin Abdulaziz College of EMS at King Saud University in Riyadh, Saudi Arabia, in October and November 2013, involving a total of 10 ambulances. The study reported a 90.00% total ambulance bacterial contamination rate (100%, 90%, and 80% contamination rates of the interior handle of the rear door, oxygen knobs, and stretcher handles, respectively) [14].

It was evident from the distribution of bacterial isolates on each ambulance site in our study that 96.1% of all the bacterial isolates were Gram-positive species. Accounting for 60.8% of all bacterial isolates, *Bacillus* spp. were the most prevalent, followed by *Micrococcus* spp., which accounted for 30.4% of all the bacterial isolates. Only 3.9% of the detected total bacterial contaminants were Gram-negative isolates. Although *Pseudomonas aeruginosa* and *Proteus* spp. isolates were sensitive to all the antimicrobials tested, many Gram-positive bacterial isolates were resistant to some antibiotics at variable frequencies. In line with our study, great variabilities in the bacterial contaminants of ambulances and their sensitivity/resistance to antimicrobials reported were in [6,7,8,9,10,11,12,13,14,15,16,17,18,19,20,21,22,23,24,25], as presented in Appendix A Table A1. The predominance of Gram-positive bacterial isolates was clear in a previous Saudi Arabian study in which *Bacillus* spp. and coagulase negative staphylococci were the top two detected bacterial contaminants [14]. *Bacillus* spp. can be transmitted via air currents, especially during hot, dry summer months [32], as in many Saudi regions. *Bacillus* spp. have been reported in other countries too, including the UK [6], the USA [7], and Spain [19].

To assess safety, microbiological standards that include the presence of indicator organisms can be implemented. Identifying these organisms helps determine potential risks to human health in HC settings. *Staphylococcus aureus* can be considered a reliable indicator of environmental hygiene [33]. While *Staphylococcus aureus* was not detected among the isolates in our study, it has been detected in the UK [6], the USA [8,11,22], Germany [9,16], South Korea [10,12], Thailand [15], Australia [17], Denmark [18,20], Spain [19], Egypt [21], India [24], and Poland [25].

The variability in the contamination rates of ambulances, their highly contaminated sites, and the types of bacterial isolates can be attributed to several factors, including the nature of emergency calls (for example, a trauma call may introduce more pathogens than non-emergency calls), patient demographics (including age and health status, as some people may carry higher bacterial loads), variations in decontamination practices (cleaning and disinfection protocols, equipment, and frequency of decontamination), and environmental conditions. Environmental factors such as urban density, pollution levels, and environmental temperature can influence the bacterial load present on surfaces. While it is conceivable that the intense heat typical of Saudi Arabia’s environment could serve as a defense against certain infections, it was clearly demonstrated in both our study and the earlier Saudi research that *Bacillus* spp. can resist hot atmospheres. The different types of materials utilized in an ambulance’s interior, which may retain bacteria to variable degrees, can also have an impact on contamination levels, as can seasonal bacterial fluctuations and high passenger turnover. These discrepancies are further exacerbated by the infrastructure of HC facilities in particular regions and the existence of microbial resistance, underscoring the necessity of using focused approaches to enhance the EMS’ infection prevention and control regulations.

The bacteria associated with HAIs possess two notable characteristics. They not only cause illness but also can persist in hospital environments for weeks. It is essential to ensure that pathogens are not present on surfaces in HC settings to maintain safety for both patients and HC workers. Important bacteria to steer clear of in these environments include Methicillin-resistant *Staphylococcus aureus* (MRSA), Vancomycin-resistant Enterococci (VRE), *Clostridium difficile*, and Acinetobacter. While Gram-negative coliforms, such as *E. coli* and *Klebsiella* spp., are not as resilient, they can survive on both dry and wet surfaces, albeit for shorter durations compared to the more durable Acinetobacter and Gram-positive bacteria mentioned earlier [32].

The results of our study show that no bacterial growth was detected in the samples taken from the ambulances immediately after they were cleaned and disinfected, suggesting that efficient infection prevention and control measures were implemented. Alqwarah General Hospital in Al-Qassim region, Saudi Arabia, implements the EMS infection control guidelines of the Saudi Ministry of health [34]. Briefly, these infection prevention and control guidelines include patient transfer recommendations; immunization programs; tuberculosis screening guidelines for EMS providers; an EMS provider fitness test and basic infection control skills license (BICSL); a patient isolation guide for EMS transport; standard precautions; hand hygiene; personal protective equipment (PPE), cleaning and disinfection for vehicle equipment, patient contact surfaces, and ambulance surfaces, as presented in Table 5 and Table 6; continuous availability of supplies; and education, training, compliance, and monitoring for EMS providers. Furthermore, the infection control practices are audited using a checklist for the cleaning and disinfection of the ambulances.

Aerobic colony counts of less than 2.5 to 5 colony-forming units per cm^2^ on surfaces frequently touched by hands are recognized as microbiological standards for surfaces inside hospitals [35]. Although achieving a completely sterile environment is unrealistic, the objective is to reduce the number of harmful microorganisms on ambulance surfaces to safeguard patients and HC providers. It is essential to set an appropriate benchmark for regular microbiological monitoring of surface cleanliness in ambulances. Once these benchmarks are in place, ongoing microbiological monitoring can reveal trends in ambulance cleanliness and workload and, most importantly, signal when additional cleaning is necessary to prevent potential infections. Adhering to best practices for cleaning and disinfecting EMS vehicles and patient care equipment is crucial for controlling the spread of infections.

Among the limitations of our study were the small sample size (204 swabs) and the fact that the swab samples were only collected from a single medical center. Furthermore, we did not evaluate seasonal bacterial fluctuations. Nonetheless, we focused on a crucial issue and think we have enough data to achieve our goal and support our findings. Despite these limitations, our work emphasizes the necessity of further investigation into the bacteria that frequently result in HAIs via ambulance environments.

## 5. Conclusions and Recommendations

To the best of our knowledge, this is the second Saudi study to evaluate the prevalence of bacterial contaminants in swab samples obtained from Saudi Arabian ambulances, and it could help health policy makers in improving the infection prevention control measures implemented in Saudi Arabian ambulances. The swab samples collected from the ambulances after the patients were transported revealed bacterial contaminants with varying degrees of reduced antimicrobial susceptibility. Ambulance environments can be sources of HAIs. No bacterial contaminants were detected after proper cleaning and disinfection. Optimal infection control measures, including effective policies for cleaning and disinfecting ambulances, can minimize the potential of bacterial infection for high-risk patients, their relatives, and HC providers. Our study underscores the need for more widespread and in-depth studies of the ambulance microbiome as an important pathogen reservoir for HAIs. It is essential to establish a suitable benchmark for routine microbiological surveillance of ambulance surface safety.

## Figures and Tables

**Table 1:** Frequencies of positive bacterial growth from each site of the ambulance vehicles based on the samples collected following patient transport.

Site	Number of Swab Samples Collected	Bacterial-Growth-Positive Samples: n. (%)	Test of Significance—Chi-Square Test (_X_^2^)	*p*
Carrying handle of cart	12	4 (33.33%)	2.667	^FE^ *p* = 0.220
Sidebar of cart	12	4 (33.33%)	2.667	^FE^ *p* = 0.220
DC shock apparatus	12	10 (83.33%)	10.667	^FE^ *p* = 0.003 *
Stethoscope	12	5 (41.67%)	0.667	0.414
Cervical collar	12	6 (50.00%)	0.000	1.000
Inside wall of the vehicle	12	5 (41.67%)	0.667	0.414
Ceiling	12	9 (75.00%)	6.000	^FE^ *p* = 0.039 *
Medical bag handles	12	5 (41.67%)	0.667	0.414
Emergency personnel seats	12	8 (66.66%)	2.667	^FE^ *p* = 0.220
Portable ventilator	12	6 (50.00%)	0.000	1.000
Blood pressure cuff	12	3 (25.00%)	6.000	^FE^ *p* = 0.039 *
Monitor	12	5 (41.67%)	0.667	0.414
Door grip	12	5 (41.67%)	0.667	0.414
Oxygen humidifier glass	12	3 (25.00%)	6.000	^FE^ *p* = 0.039 *
Suction device	12	5 (41.67%)	0.667	0.414
Headboard of patient stretcher	12	5 (41.67%)	0.667	0.414
Steering wheel	12	6 (50.00%)	0.000	1.000
THE SUM	204	94 (46.08%)	-	-

^FE^: Fisher Exact. _X_^2^: Chi-square test. * The result is statistically significant at *p* ≤ 0.05. Statistically, it is clear that DC shock apparatuses and ceilings have a greater probability of being contaminated. In contrast, blood pressure cuffs and oxygen humidifier glass have a greater likelihood of being noncontaminated.

**Table 2:** Frequencies of bacterial isolates in each site of ambulances based on the samples collected following patient transport.

Site	Positive Bacterial Growth	
	Number of Bacterial Isolates	Percentage (Number of Bacterial Isolates/102 Isolates)
Carrying handle of cart	4	3.9%
Sidebar of cart	5	4.9%
DC shock apparatus	10	9.8%
Stethoscope	5	4.9%
Cervical collar	7	6.8%
Inside wall of the vehicle	5	4.9%
Ceiling	11	10.8%
Medical bag handles	5	4.9%
Emergency personnel seats	8	7.8%
Portable ventilator	6	5.9%
Blood pressure cuff	3	2.9%
Monitor	7	6.8%
Door grip	6	5.9%
Oxygen humidifier glass	3	2.9%
Suction device	5	4.9%
Headboard of patient stretcher	6	5.9%
Steering wheel	6	5.9%
THE SUM; n. (%)	102	100.0%

**Table 3:** Distribution of bacterial isolates on each site in the ambulances, as derived from the samples collected following patient transport.

Site	*Bacillus* spp. n.	*Micrococcus* spp. n.	*Moraxella* spp. n.	*Staphylococcus epidermidis * n.	*Staphylococcus saprophyticus* n.	*Proteus* spp. n.	*Pseudomonas aeruginosa* n.	*Streptococcus viridans * n.
Carrying handle of cart	2	2	0	0	0	0	0	0
Sidebar of cart	2	2	0	0	1	0	0	0
DC shock apparatus	8	0	1	0	0	0	0	1
Stethoscope	5	0	0	0	0	0	0	0
Cervical collar	6	1	0	0	0	0	0	0
Inside wall of the vehicle	2	3	0	0	0	0	0	0
Ceiling	7	3	0	1	0	0	0	0
Medical bag handles	3	1	0	0	1	0	0	0
Emergency personnel seats	5	3	0	0	0	0	0	0
Portable ventilator	3	3	0	0	0	0	0	0
Blood pressure cuff	3	0	0	0	0	0	0	0
Monitor	4	0	1	0	0	1	1	0
Door grip	3	3	0	0	0	0	0	0
Oxygen humidifier glass	1	2	0	0	0	0	0	0
Suction device	2	3	0	0	0	0	0	0
Headboard of patient stretcher	4	2	0	0	0	0	0	0
Steering wheel	2	3	0	1	0	0	0	0
Frequency; n. (%)	62/102 (60.8%)	31/102 (30.4%)	2/102 (1.96%)	2/102 (1.96%)	2/102 (1.96%)	1/102 (0.98%)	1/102 (0.98%)	1/102 (0.98%)

**Table 4:** Antimicrobial resistance patterns of Gram-positive bacterial isolates, as derived via disc diffusion method. Data shown are frequencies (n (%)).

Antibiotic	*Bacillus* spp. n. 62 (60.8%)	*Micrococcus* spp. n. 31 (30.4%)	*Staphylococcus epidermidis * n. 2 (1.96%)	*Staphylococcus saprophyticus* n. 2 (1.96%)	*Streptococcus viridans * n. 1 (0.98%)
**AP:** Ampicillin 10 μg	1 (1.6%)	0 (0.0%)	0 (0.0%)	0 (0.0%)	0 (0.0%)
**AK**: Amikacin 30 μg	0 (0.0%)	0 (0.0%)	0 (0.0%)	0 (0.0%)	0 (0.0%)
**AUG:** Augmentin 30 μg	1 (1.6%)	0 (0.0%)	0 (0.0%)	0 (0.0%)	0 (0.0%)
**BA:** Bacitracin 10 U	14 (22.6%)	2 (6.5%)	0 (0.0%)	0 (0.0%)	0 (0.0%)
**C:** Chloramphenicol 30 μg	0 (0.0%)	0 (0.0%)	0 (0.0%)	0 (0.0%)	0 (0.0%)
**CAZ**: Ceftazidime 30 μg	1 (1.6%)	0 (0.0%)	0 (0.0%)	0 (0.0%)	0 (0.0%)
**CIP**: Ciprofloxacin 5 μg	0 (0.0%)	0 (0.0%)	0 (0.0%)	0 (0.0%)	0 (0.0%)
**CD:** Clindamycin 2 μg	0 (0.0%)	0 (0.0%)	0 (0.0%)	0 (0.0%)	0 (0.0%)
**CPM:** Cefepime 30 μg	0 (0.0%)	1 (3.2%)	0 (0.0%)	1 (50.0%)	0 (0.0%)
**E:** Erythromycin 15 μg	0 (0.0%)	4 (12.9%)	1 (50.0%)	0 (0.0%)	0 (0.0%)
**FOX:** Cefoxitin 30 μg	1 (1.6%)	1 (3.2%)	0 (0.0%)	0 (0.0%)	0 (0.0%)
**GM:** Gentamicin 10 μg	1 (1.6%)	2 (6.5%)	0 (0.0%)	0 (0.0%)	1 (100.0%)
**IPM:** Imipenem 10 μg	0 (0.0%)	0 (0.0%)	0 (0.0%)	0 (0.0%)	0 (0.0%)
**KF:** Cephalothin 30 μg	4 (6.5%)	2 (6.5%)	1 (50.0%)	0 (0.0%)	1 (100.0%)
**NE:** Neomycin 30 μg	1 (1.6%)	1 (3.2%)	0 (0.0%)	0 (0.0%)	1 (100.0%)
**OT:** Oxytetracycline 30 μg	0 (0.0%)	1 (3.2%)	0 (0.0%)	0 (0.0%)	1 (100.0%)
**OX:** Oxacillin 5 μg	3 (4.8%)	3 (9.7%)	2 (100.0%)	1 (50.0%)	1 (100.0%)
**PG:** Penicillin G 10 U	31 (50.0%)	11 (35.5%)	1 (50.0%)	2 (100.0%)	1 (100.0%)
**PRL**: Piperacillin 30 μg	0 (0.0%)	0 (0.0%)	0 (0.0%)	0 (0.0%)	0 (0.0%)
**T:** Tetracycline 30 μg	0 (0.0%)	0 (0.0%)	0 (0.0%)	0 (0.0%)	1 (100.0%)
**TS:** Cotrimoxazole 25 μg	11 (17.7%)	5 (16.1%)	0 (0.0%)	2 (100.0%)	1 (100.0%)
**VA:** Vancomycin 30 μg	0 (0.0%)	0 (0.0%)	0 (0.0%)	0 (0.0%)	0 (0.0%)

**Table 5:** Recommendations for ambulance decontamination (cleaning and disinfection) following patient transport according to the Saudi Ministry of Health infection control guidelines [34].

Site	Standard	Decontamination Frequency	Additional Considerations
Carrying handle of cart	It must be visibly clean and free of dust, dirt, stains, blood, bodily fluids, or any spillages.	After each patient care procedure.	-
Sidebar of cart	It must be visibly clean and free of dust, dirt, stains, blood, bodily fluids, or any spillages.	After each patient care procedure.	-
DC shock apparatus	All parts must be visibly clean and free of dust, dirt, stains, blood, bodily fluids, or any spillages.	After each patient use.	-
Stethoscope	It must be visibly clean and free of dust, dirt, stains, blood, bodily fluids, or any spillages.	After each patient use.	-
Cervical collar	All parts must be visibly clean and free of dust, dirt, stains, blood, bodily fluids, or any spillages.	After each patient use.	-
Inside wall of the vehicle	All wall surfaces must be visibly clean and free of dust, dirt, stains, blood, bodily fluids, adhesive tape, or any spillages.	Every week.	If contaminated, decontaminate it as soon as possible before further patient transfer.
Ceiling	All surfaces must be visibly clean and free of dust, dirt, stains, blood, bodily fluids, adhesive tape, or any spillages.	Every week.	If contaminated, decontaminate it as soon as possible before further patient transfer.
Response kits and medical bags	All parts of medical bags must be visibly clean and free of dust, dirt, blood, bodily fluids, or any spillages.	Bags frequently brought into patient care areas must be decontaminated after each use, especially if they are contaminated with blood or bodily fluids. Heavily used bags should be laundered weekly or monthly, while those used less frequently should be cleaned every other month.	All bags used in ambulances should be made from materials that can be easily wiped clean. Any bag that is heavily contaminated with blood or bodily fluids should be disposed of.
Emergency personnel seats	The cover must be free of damage. All components, including seatbelts and the areas underneath, should be visibly clean and free from dust, dirt, stains, blood, bodily fluids, or any spillages.	After each use.	Replace seatbelts if they are heavily contaminated with blood or bodily fluids. Torn or damaged seat covers should also be replaced.
Portable ventilator	All components, including the valve and cylinder, must be visibly clean and free from dust, dirt, stains, blood, bodily fluids, or any spillages.	After each use.	Single-use items should be replaced after each use.
Blood pressure cuff	All parts must be visibly clean and free of dust, dirt, stains, blood, bodily fluids, or any spillages.	After each patient use.	-
Monitor	All parts must be visibly clean and free of dust, dirt, blood, bodily fluids, or any spillages.	After each patient use.	-
Door grip	It must be visibly clean and free of dust, dirt, stains, blood, bodily fluids, or any spillages.	After each patient use.	-
Oxygen humidifier glass	All parts, including the valve and cylinder, must be visibly clean and free of dust, dirt, stains, blood, bodily fluids, or any spillages.	After each use.	Single-use items should be replaced after each use.
Suction device	All parts, including the valve and cylinder, must be visibly clean and free of dust, dirt, stains, blood, bodily fluids, or any spillages.	After each use.	Single-use items should be replaced after each use.
Stretcher	All parts must be visibly clean and free of dust, dirt, blood, bodily fluids, or any spillages.	After each patient use.	-
Driver’s compartment	All areas of the driver’s compartment must be visibly clean and free from dust, dirt, debris, blood, bodily fluids, or any spillages.	After every patient care procedure, if the driver participated in direct patient care, such as moving the patients on stretchers. After completing the direct patient care procedure and before entering the driver’s compartment, drivers should remove PPE (except masks/respirators) and ensure hand hygiene to avoid contamination of the driver’s compartment.	If possible, employ cars with separate driver and patient compartments that allow for separate ventilation. Before bringing the patient aboard, close the door or window between these compartments. Clean and vacuum the floor.

**Table 6:** Recommended ambulance disinfectants according to the Saudi Ministry of Health infection control guidelines [34].

Products	Disadvantage	Advantages	Uses
Standard Bleach (Normal dilution 1:10)	It damages metals and can be deactivated by organic materials, necessitating a clean surface before use. It can stain clothing. It can also irritate mucous membranes and skin, and once diluted, it should be used within 24 h.	It is inexpensive, acts quickly, and is easily accessible. It comes in convenient, ready-to-use wipes and sprays and is both viricidal and sporicidal, making it effective against Norovirus as well as spore-forming bacteria like *Clostridia* and *Bacillus* spp.	- Blood spills - External surfaces
Alcohol (70–95%)	It evaporates quickly and is not the best choice for surface disinfection. It is highly flammable and can damage plastic, silicone, and rubber. Additionally, it is rendered ineffective by organic materials, so surfaces must be cleaned before use.	It is non-toxic, affordable, acts quickly, and leaves no residue.	External surfaces of certain pieces of equipment, including stethoscopes and pulse oximeters.
Quaternary ammonium compounds	They are unsuitable for disinfecting medical instruments and have limited application potential as disinfectants due to their narrow range of effectiveness against microbes.	They are non-toxic and non-corrosive and possess effective cleaning properties thanks to their detergent properties.	- Walls, floors, and furnishings - Blood spills, prior to disinfection
Hydrogen Peroxide	It damages materials such as copper, zinc, brass, acrylics, and aluminum. It also leaves a visible residue behind.	It is environmentally safe and non-toxic and acts quickly while remaining effective even in the presence of organic substances. It is available in both wipes and liquid form and offers excellent cleaning capabilities due to its excellent detergent properties.	- Walls, floors, and furnishings - External surfaces of some pieces of equipment

## Data Availability

The original contributions presented in this study are included in the article. Further inquiries can be directed to the corresponding authors.

## Appendix A

**Table A1:** Previous studies reporting bacterial contamination in ambulances.

Country	Publication Year	Collection of Samples	Detected Organism(s)	Contamination Frequency	Reported Highly Contaminated Surfaces	Reference No.
**UK**	2003	12 ambulances (7 surfaces).	*Bacillus* sp., coliforms, *Micrococcus* sp., *Corynebacterium* sp., *Pseudomonas* sp., *S. viridans*, *S. aureus*, *S. epidermidi*	various bacteria: 61%. *S. viridans*: 2.7%. *S. aureus*: 1.4%.	Rails of grids/tracks or floor, inside of suction bottle, folds in stretcher mattress, offside wall near stretcher mattress, inside mask (Entonox).	[6]
**USA**	2006	One air ambulance (helicopter, 7 surfaces).	*Staphylococcus* sp., *Pseudomonas* sp., *E. coli*, *Bacillus* sp., *Aspergillus* sp.	*Staphylococcus* sp.: 6/7 samples (86%). *Pseudomonas* sp.: 2/7 samples (29%)	Door handles, seat tracks, stretcher.	[7]
**USA**	2010	51 ambulances (16 surfaces).	MRSA.	MRSA detected in 50% of ambulances (25/51): Fire Department ambulances (25/51), with 56% (14/25) contaminated. Private nonprofit ambulances (19/51), with 45% (9/19) contaminated. Hospital-based ambulances (5/51), with 20% (1/5) contaminated. Third-service municipal ambulances (2/51), with 50% (1/2) contaminated.	Stretcher rail, work area beside patient, pulse oximeter.	[8]
**Germany**	2011	89 transport events with MRSA notification and 60 transport events without MRSA notification (5 surfaces).	MRSA, MSSA	MRSA was found in 8 out of 89 transport events, representing 9% of transports with an MRSA notification. The distribution is as follows: stretcher headrest—5 out of 8 (63%); stretcher handles—2 out of 8 (25%); both headrest and stretcher handles—1 out of 8 (12%). MSSA was identified in 12 out of 60 transport events, accounting for 20% of transports without MRSA notification. The distribution is as follows: stretcher headrest—5 out of 12 (42%); stretcher handles—6 out of 12 (50%); cabin wall—1 out of 12 (<1%).	Stretcher headrest and handles.	[9]
**South Korea**	2011	13 ambulances (33 surfaces).	*K. pneumoniae* ESBL, *P. aeruginosa*, MRCoNS, MRSA. ^a^	All ambulances showed microbial contamination. from 429 total surfaces, 396/624 (64%) sample cultures positive for organisms. Two *K. pneumoniae* ESBL from water in suction bottle, and bag-valve mask bag. One *P. aeruginosa.* One MRCoNS from stretcher car side bar. One MRSA from driver’s side door handle.	Water from suction bottles, stretcher car side bars, bag-valve mask bag, driver’s side door handle.	[10]
**USA**	2012	71 ambulances (26 surfaces).	MRSA, MSSA.	Out of 71 ambulances, 49 had at least one isolate of *S. aureus*, with 100 out of 1,125 isolates identified as *S. aureus*. Among these isolates, 77% were resistant to ≥one antibiotic, and 34% were resistant to ≥two antibiotics. Regarding MRSA, 5 out of 71 ambulances tested positive, with 9 out of 26 surfaces showing at least one MRSA isolate, totaling 12 isolates. For MSSA, 49 out of 71 ambulances had isolates, with 22 out of 26 surfaces containing at least one MSSA isolate, resulting in a total of 88 isolates.	Portable pulse oximetry finger sensors, automatic pulse finger sensors, portable pulse oximeter outer case, workspace deck, automatic blood pressure cuffs.	[11]
**South Korea**	2012	30 ambulances (33 surfaces).	*S. aureus*, *P. aeruginosa*, *Serratia marcescens*, *Legionella* spp. ^a^	All ambulances tested positive for contamination on at least 3 surfaces, with 159 out of 955 surfaces found to be contaminated. A total of 28 bacterial species were isolated, resulting in 184 total isolates of pathogenic bacteria (14 out of 184, or 8%): *S. aureus* accounted for 7 out of 184 isolates (8%) and was found on the endotracheal tube and suction tip. *P. aeruginosa* accounted for 4 out of 184 isolates (2%); it was isolated from the suction tip, oropharyngeal airway, and oxygen humidifier water. *S. marcescens* represented 2 out of 184 isolates (1%) and was retrieved from the endotracheal tube. Legionella was detected in 1 out of 184 isolates (0.5%), deriving from suction water.	100% endotracheal tube, 90% oxygen humidifier (water), 80% water from suction reservoirs.	[12]
**Iraq**	2013	139 ambulances, including air ambulances (6 surfaces).	*Pantoea agglomerans*, *Shigella flexneri*, *Pseudomonas* spp., *Escherichia vulneris*, *K. pneumoniae*.	In 13 out of 139 ambulances, 79 out of 134 swabs revealed at least one Gram-negative colony-forming unit. Among the isolated organisms, *P. agglomerans* accounted for 34%, while *S. flexneri* represented 8%. Additionally, *Pseudomonas* spp., *E. vulneris*, and *K. pneumoniae* each comprised 6% of the isolates.	Door handles, panels, steering wheels, electronic equipment, stretchers, floors.	[13]
**Saudi Arabia**	2014	10 ambulances (3 surfaces).	*Bacillus* sp., CoNS. ^a^	Contamination was observed on three surfaces before and after the fumigation of the ambulance. The oxygen knob showed a contamination occurrence of 9 before fumigation and 1 after, while the stretcher handle had 8 before and 1 after. The interior handle of the rear door presented contamination levels of 10 before fumigation and 4 afterward.	100% interior handle of rear door, 90% oxygen knob, 80% stretcher handle.	[14]
**Thailand**	2015	30 ambulances (318 air samples).	*S. aureus*, *Aspergillus* sp., *Fusarium* sp., *Penicillium* sp.	*S. aureus* (47/91 samples; 51.6%).	Stretchers, air-flow fins, stethoscope, oxygen flow knob.	[15]
**Germany**	2015	150 ambulances (28 surfaces).	*S. aureus*, MRSA, CoNS. ^a^	11/150 ambulances; 10/28 surfaces. MRSA was detected on 18 plates.	Carrying handles, oxygen saturation clip, patient stretcher handle, cardiovascular bag handle, patient stretcher headboard, BP cuff, pharmacist cabinet handle, carrying chair, ECG control panel.	[16]
**Australia**	2016	2 Air ambulances (helicopters; 5 surfaces).	MSSA, *S. epidermidis*.	MSSA (19/60 samples; 32%).	Floor of helicopters, seat, blood pressure cuff containers.	[17]
**Denmark**	2016	39 ambulances (1 surface).	*S. aureus*, Enterococcus.	Out of 50 blood pressure cuffs examined, including 11 duplicate cuffs, contamination was found in 6. Specifically, *S. aureus* was identified in 5 of the cuffs, accounting for 10%, while Enterococcus was present in 1 cuff, representing 2%.	Blood pressure cuffs.	[18]
**Spain**	2017	10 ambulances (4 surfaces).	CoNS, MSSA, Enterobacterales, nonfermenting Gram-negative bacilli, Enterococci, *Bacillus* spp. ^a^	Contamination was detected on 26 out of 40 surfaces in the patient compartment. Among the isolates, CoNS were found in 15 out of 44 instances, representing 34%. Additionally, non-fermenting Gram-negative bacilli, Enterobacterales, Enterococci, and *Bacillus* spp. were identified in 26 out of 44 cases, accounting for 59%.	Interior passenger door handle, left handle of stretcher, steering wheel.	[19]
**Denmark**	2018	80 ambulances (6 surfaces).	*S. aureus* including MRSA, Enterococcus including VRE, Enterobacterales.	Contamination was found on 49 out of 480 ambulance surfaces, with *S. aureus* detected in 7% of cases and Enterobacterales in 1%. In total, 108 sites tested positive for pathogens, including two instances of VRE and one case of MRSA.	Blood pressure cuffs, medical bag handles, patient harness.	[20]
**Egypt**	2018	25 ambulances (16 surfaces).	*Staphylococcus* spp. including MRSA and MRCoNS, *Klebsiella pneumoniae* including *K. pneumoniae* ESBL, *Escherichia coli* including *E. coli* ESBL, *Citrobacter* spp., *Proteus* spp. ^a^	In all ambulances, contamination was identified on 400 out of 400 surfaces. A total of 184 staphylococcal isolates were found, with MRSA accounting for 35 out of 184 isolates, representing 19%, and MRCoNS comprising 22 out of 184 isolates (12%). Additionally, there were 49 isolates of *K. pneumoniae*, with 18 out of those 49 being ESBL producers (37%). Furthermore, 40 isolates of *E. coli* were identified, and among those, 11 out of 40 were classified as ESBL producers (3%).	Headboard of patient stretcher, suction devices, beds.	[21]
**USA**	2019	3 ambulances (13 surfaces).	MRSA.	In all ambulances, contamination was detected on 5 out of 39 surfaces, with MRSA identified in 13 out of 39 instances (33%). Specifically, all 9 oxygen cylinders showed contamination, averaging 3 per ambulance, while contamination was found on the patient compartment floor in all 3 cases and on the rear door handle in 1 out of 3 instances.	Oxygen cylinders.	[22]
**Iran**	2020	12 ambulances (18 surfaces).	CoNS, *Klebsiella* spp., *Acinetobacter* spp., *Pseudomonas* spp., *Proteus* spp., *Corynebacterium diphtheriae*.	All ambulances and all surfaces (100%). CoNS, 76 isolates. Klebsiella spp, 8 isolates. *Acinetobacter* spp., 6 isolates. *Pseudomonas* spp., 6 isolates. *Proteus* spp., 4 isolates. *C. diphtheriae*, 2 isolates.	Oxygen tank.	[23]
**India**	2021	5 ambulances (22 surfaces).	*S. aureus*, *Klebsiella* spp., *Escherichia coli*, *Proteus* spp., *Enterococcus* spp., *Pseudomonas aeruginosa*.	Out of 198 swabs, 170 (85.8%) were found to be sterile, while 28 swabs (14.2%) yielded bacterial isolates. Among these isolates, *S. aureus* accounted for 32%, with MRSA making up 22% of the *S. aureus* isolates. Additionally, *Klebsiella* spp. comprised 21.4%, *E. coli* represented 14.2%, and *Proteus* spp. also accounted for 14.2%. *Enterococcus* spp. contributed 10.7%, while *P. aeruginosa* represented 7.2% of the isolates.	Oxygen flow meter knob (60%), suction machine tubing (60%), stethoscope (40%).	[24]
**Poland**	2024	One ambulance (20 surfaces).	MRSA, MSSA.	20 (51.28%) swabs yielded *S. aureus*, of which MRSA represented 40% (8/20 swabs), and MSSA represented 60% (12/20 swabs).	The highest contamination rates for MRSA were found on surfaces such as the tourniquet, pulse oximeter, stethoscope, glucometer, defibrillator panels with buttons, and door handles. In contrast, the surfaces with the highest rates of MSSA included the pressure stasis device, blood pressure cuff, steering wheel, ECG cables, and defibrillator screens and panels with buttons, as well as the stretcher’s head support and mattress.	[25]

BP, blood pressure; CoNS, coagulase-negative Staphylococci; ECG, electrocardiogram; *E. coli*, *Escherichia coli*; ESBL, extended-spectrum b-lactamase; *K. pneumoniae*, *Klebsiella pneumoniae*; MRCoNS, methicillin-resistant coagulase-negative Staphylococci; MRSA, methicillin-resistant *Staphylococcus aureus*; MSSA, methicillin-sensitive *Staphylococcus aureus*, *P. aeruginosa*, *Pseudomonas aeruginosa*; *S. aureus*, *Staphylococcus aureus*; *S. viridans*, *Streptococcus viridans*; VRE, vancomycin-resistant Enterococci. ^a^ Various other bacteria and/or fungi were detected.
